# Spectroscopic-ellipsometric study of native oxide removal by liquid phase HF process

**DOI:** 10.1186/2228-5326-3-10

**Published:** 2013-02-22

**Authors:** Anil Sudhakar Kurhekar, Prakash R Apte

**Affiliations:** 1 Department of Electrical Engineering, Indian Institute of Technology Bombay, Powai, Mumbai, India; 2 Department of Electronics Engineering, Datta Meghe College of Engineering, Airoli, Navi, Mumbai, India

**Keywords:** Silicon<100>, Spectroscopy, Ellipsometry, Native oxide removal, FTIR, Cleavage, MEMS

## Abstract

*Ex situ* spectroscopic ellipsometry (SE) measurements have been employed to investigate the effect of liquid-phase hydrofluoric acid (HF) cleaning on Si<100> surfaces for microelectromechanical systems application. The hydrogen terminated (H-terminated) Si surface was realized as an equivalent dielectric layer, and SE measurements are performed. The SE analyses indicate that after a 20-s 100:5 HF dip with rinse, the Si (100) surface was passivated by the hydrogen termination and remained chemically stable. Roughness of the HF-etched bare Si (100) surface was observed and analyzed by the *ex-situ* SE. Evidence for desorption of the H-terminated Si surface layer is studied using Fourier transform infrared spectroscopy and ellipsometry, and discussed. This piece of work explains the usage of an *ex situ*, non-destructive technique capable of showing state of passivation, the H-termination of Si<100> surfaces.

## Background

**Figure 1:**
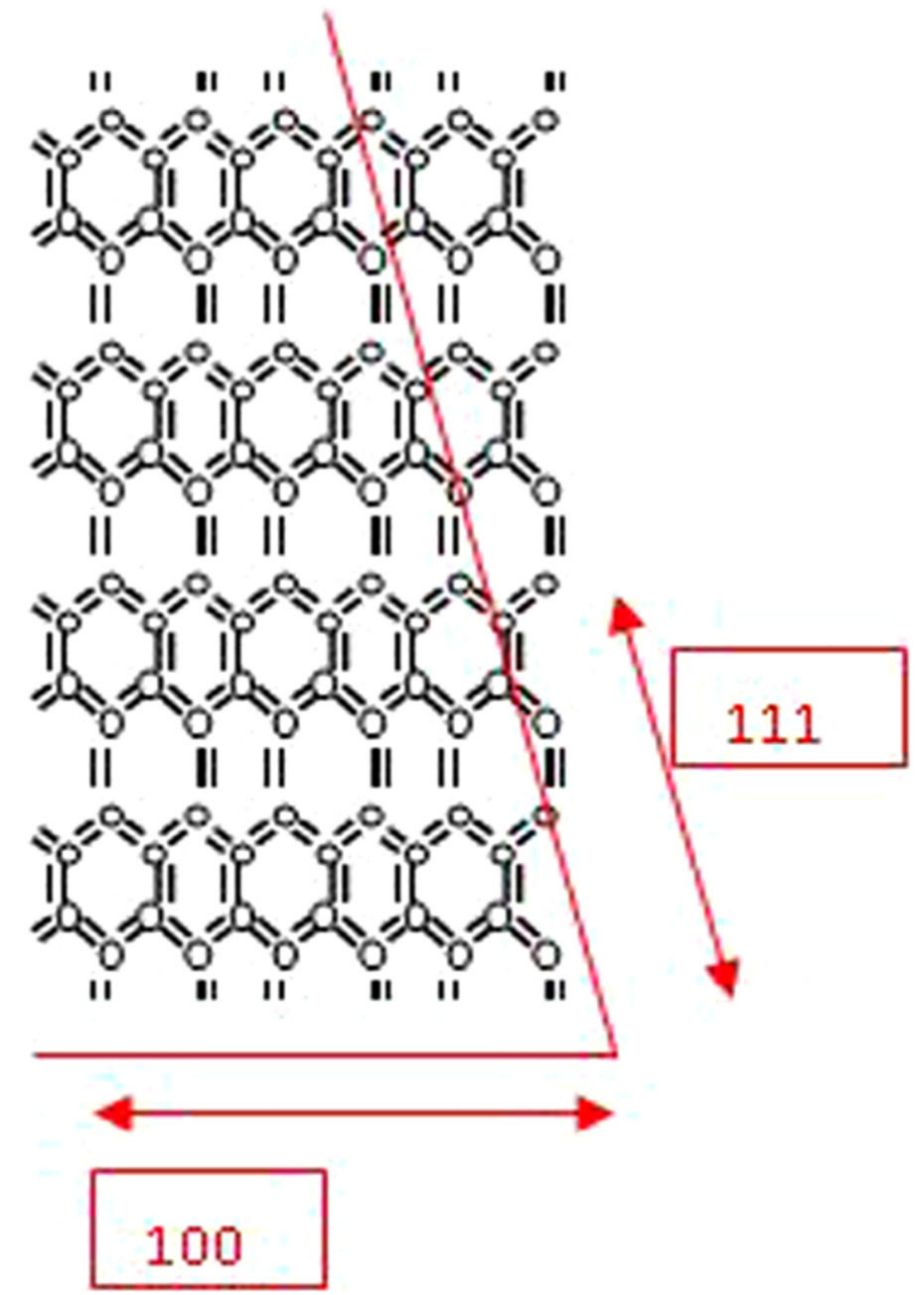
Tetrahedral arrangement of bulk structure of Si atoms.

A (100) orientation leads to a more open lattice structure, which is easy to cleave and etch chemically. The bulk structure of Si represents the tetrahedral arrangement of atoms (Figure 1).

**Figure 2:**
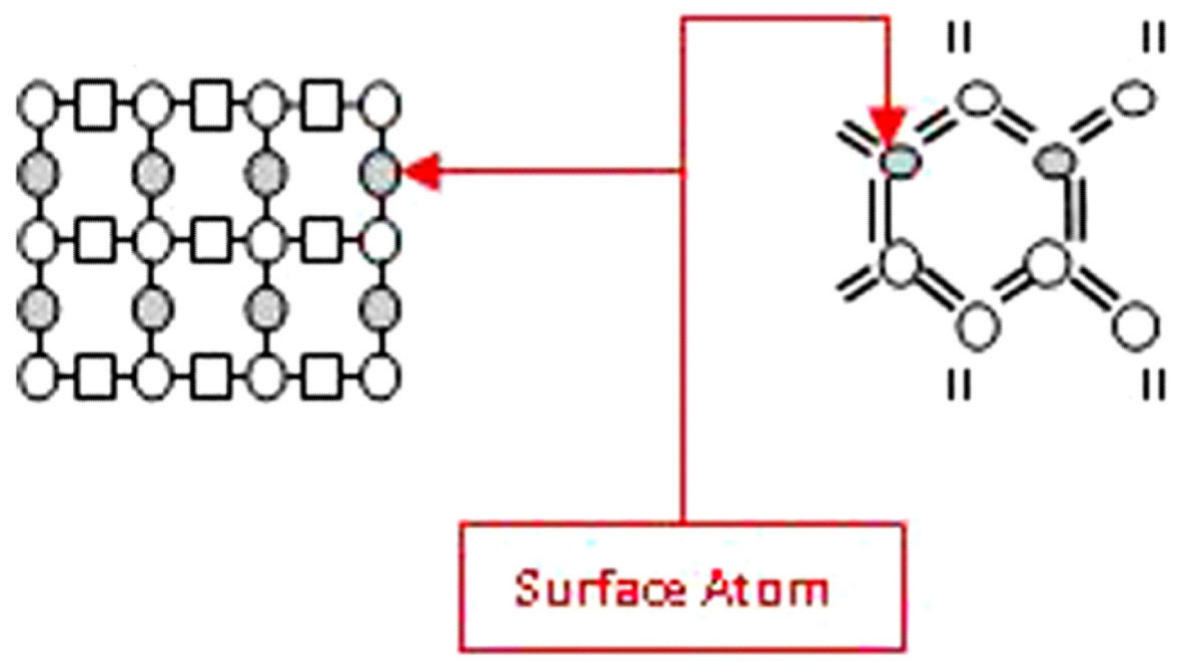
Surface arrangement of bulk structure of Si atoms.

The smaller circles represent atoms that are pointing away from the page. It can be clearly seen that the surface atoms for (111) and (100) orientation contain one and two dangling bonds, respectively. The hydride-terminated surfaces are reported to be reasonably stable and can be prepared and manipulated in air as well as in a number of organic solvents (Figure 2).

Therefore, quality materials are available without the need of expensive vacuum systems. Other advantages offered by this surface are excellent chemical homogeneity (>99% H termination) and strong Fourier transform infrared spectroscopy (FTIR) vibrational modes (Si-H stretching, *ν* = 2,100 cm^−1^), which also provide information on surface flatness.

Despite the Si-H-terminated surface for many applications [1-4] is precluded due to its propensity to oxidize, it can be easily handled in air for 1.5 h without measurable degradation. The removal of SiO_2_ by a liquid phase hydrofluoric acid (HF) [3,5-9], often referred to as ‘HF dip’, and is a very common process in silicon processing. The native chemical oxides [6] on bare silicon wafer are different from wet chemically grown oxide because of the oxygen bound to the silicon surface. Native oxide [3,10,11] has siloxane rings which are very stable against hydrolysis, rendering the surface highly hydrophobic; 1:20 vol/vol HF-water mixture [12] 1.1–1.2. Mole treatment etches the hydrophobic native SiO_2_ layer following the reaction, as denoted by the following chemical relation and leaves the surface hydrogen passivated.

(1) $$\begin{array}{cc} {\mathrm{SiO}}_{2} & \left(\text{Solid}\right)\\ + & 6\mathrm{HF}(\text{Liquid})↔H_{2}(\text{Vapor})\\ + & {\mathrm{SiF}}_{6}(\text{Liquid})\;+\;2H_{2}O(\text{liquid}) \end{array}$$

### Silicon<100> surface study

#### Ellipsometry

Ellipsometry measures a change in polarization as light reflects or transmits from a material structure [13-15]. The polarization change is represented as an amplitude ratio, Ψ, and the phase difference, Δ. The measured response depends on the optical properties and thicknesses of individual materials. Thus, ellipsometry is primarily used to determine the film thickness and optical constants. However, it is also applied to characterize composition, crystallinity, roughness, doping concentration, and other material properties associated with a change in optical response. Ellipsometry is primarily interested on how *p*- and *s*- components change upon reflection or transmission in relation to each other. In this manner, the reference beam is part of the experiment. A known polarization is reflected or transmitted from the sample, and the output polarization is measured. The change in polarization is the ellipsometry measurement, commonly written as:

(2) $$\rho =\tan(\psi )e\psi^{i\Delta }$$

**Figure 3:**
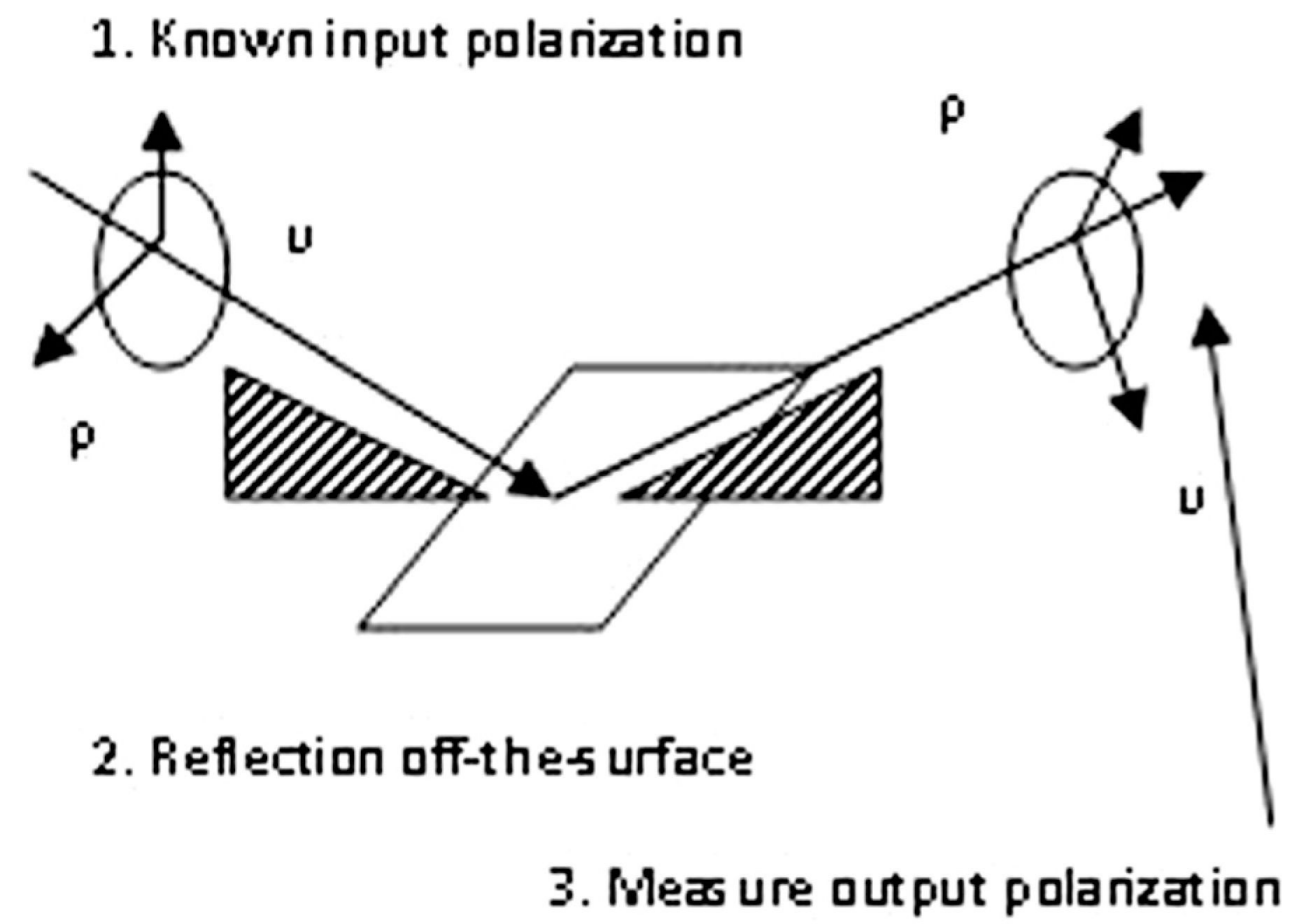
Ellipsometry.

A sample ellipsometry measurement is shown in Figure 3. The incident light is linear with both p- and s- components. The reflected light has undergone amplitude and phase changes for both p- and s- polarized light, and ellipsometry measures their changes.

In the case of a bulk material, the equations derived for a single reflection can be directly inverted to provide the ‘pseudo’ optical constants from the ellipsometry measurement, *r*:

(3) $$<\tilde{\varepsilon }1>={\mathrm{Sin}}^{2}\varphi \left[1+\tan^{2}\varphi {\left(\frac{1-\rho }{1+\rho }\right)}^{2}\right]$$

#### Film thickness

The film thickness is determined by interference between the light reflecting from the surface and the light travelling through the film. Depending on the relative phase of the rejoining light to the surface reflection, interference can be defined as constructive or destructive. The interference involves both amplitude and phase information. The phase information from Δ is very sensitive to films below the sub-monolayer thickness.

##### Optical constants

The optical constants [14-18] for a material will vary for different wavelengths and must be described at all wavelengths probed with the ellipsometer. A table of optical constants can be used to predict the material’s response at each wavelength. However, it is not very convenient to adjust unknown optical constants on a wavelength-by-wavelength basis. It is more advantageous to use all wavelengths simultaneously. A dispersion relationship often solves this problem, by describing the optical constant shape versus the wavelength. The adjustable parameters of the dispersion relationship allow the overall optical constant shape to match the experimental results. Compared to the fitting individual *n, k* values at every wavelength, this greatly reduces the number of unknown ‘free’ parameters. For transparent materials, the index is often described using the Cauchy or Sellmeier relationship. The Cauchy relationship is typically given as:

(4) $$n(\lambda )\;=\;A\;+\;\frac{B}{\lambda^{2}}+\frac{C}{\lambda^{4}}$$

where the three terms are adjusted to match the refractive index for the material. The Cauchy is not constrained by Kramers-Kronig (KK) consistency and can produce unphysical dispersion. The Sellmeier relationship enforces the KK consistency, which ensures that the optical dispersion retains a realistic shape. The Sellmeier relationship can be written as:

(5) $$\varepsilon_{1}\;=\;\frac{A\lambda^{2}\lambda_{0}^{2}}{\left(\lambda^{2}-\lambda_{0}^{2}\right)}$$

**Figure 4:**
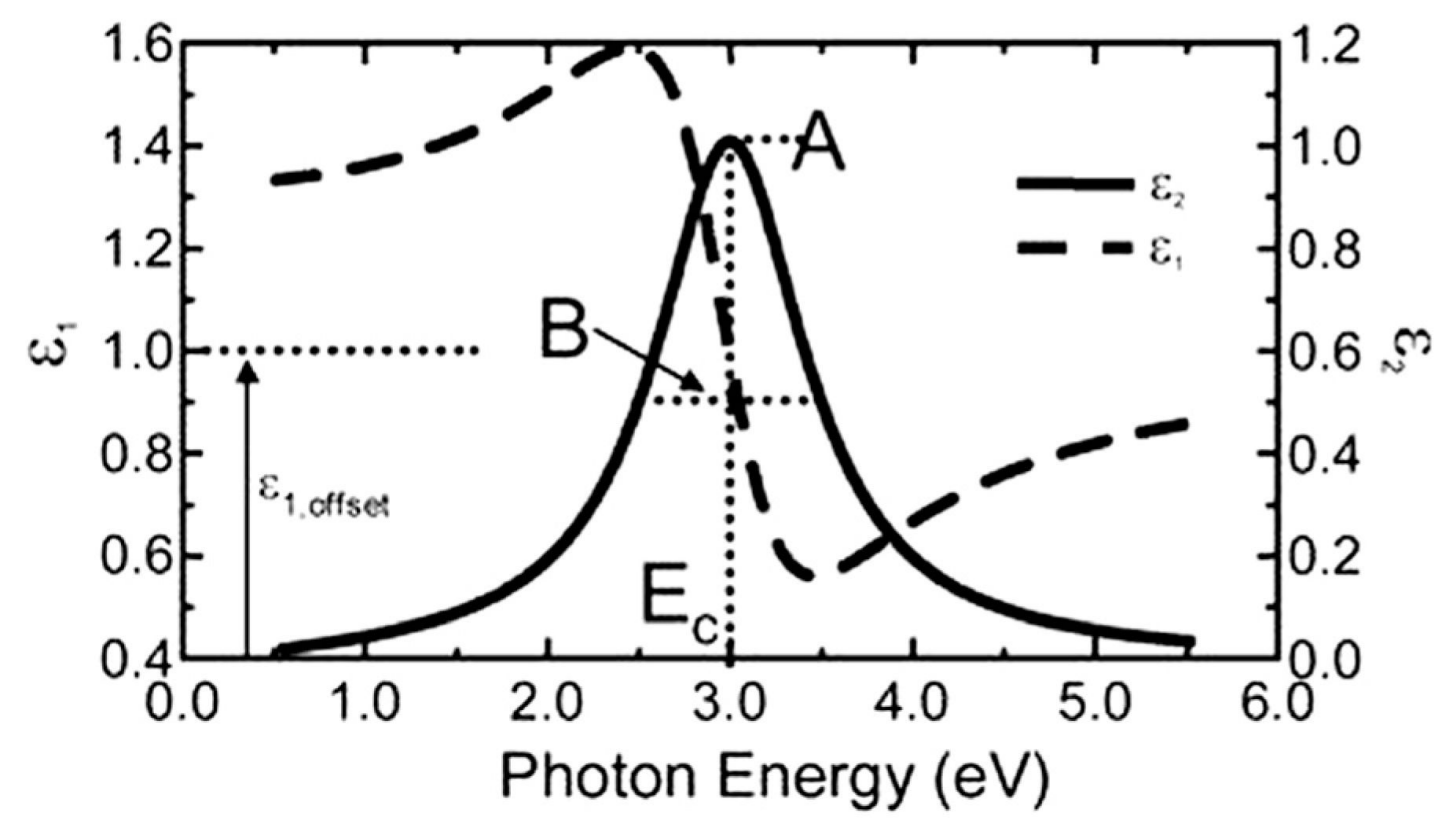
Photon energy vs. *ε*1 and *ε*2.

Absorbing materials will often have a transparent wavelength region that can be modeled with the Cauchy or Sellmeier. However, the absorbing region accounts for both real and imaginary optical constants. Mainly, oscillator theory is used in the dispersion relationships to describe absorption for various materials, which include the Lorentz, Harmonic, and Gaussian oscillators. They all depict the absorption features described with an amplitude, broadening, and center energy (related to the frequency of light). Kramers-Kronig consistency is used to calculate the shape of the real component after the imaginary behavior is described by the oscillator. An offset to the real component is added to account for extra absorption outside the measured spectral region. The Lorentz oscillator can be written as:

(6) $$\varepsilon \;=\;{\tilde{\varepsilon }}_{1,\mathrm{offset}}\;+\;\frac{AEc}{E_{c}^{2}-E^{2}-\mathrm{iBE}}$$

where the parameters for amplitude (*A*), broadening (*B*), center energy (*E*c), and offset (*ε*_1_, off set) are also shown in Figure 4 for a typical Lorentz oscillator. The energy, *E*, is related to the frequency of a wave, *n*:

(7) $$E\;=\;h\nu \approx \frac{1240}{\lambda_{\mathrm{nm}}}$$

where *h* is the Planck’s constant and the wavelength, *l*, is given in nanometers. More advanced dispersion models, like the Tauc-Lorentz and Cody-Lorentz, will include terms to describe the band-gap energy (Figure 4).

##### Infrared spectrum

An infrared spectrum represents a fingerprint of a sample with absorption peaks which correspond to the frequencies of vibrations between the bonds of the atoms making up the material. Because each different material is a unique combination of atoms, no two compounds produce exactly the same infrared spectra. Therefore, infrared spectroscopy results in a positive identification (qualitative analysis) of every different kind of material. In addition, the size of the peaks in the spectrum is a direct indication of the amount of material present.

##### Surface roughness

The rigorous coupled-wave analysis (RCWA) conventionally deals with structures consisting of a single material. However, surfaces are, in general, coated with other materials, such as oxides. Since the roughness on the layer can also be treated as an overlayer by means of the effective medium approximation (EMA) theory, we can determine the roughness by extending the RCWA to structures consisting of two or more materials.

The optical properties of composite materials can often be approximated using a uniform effective medium when the length scales associated with the local variations in the permittivity are small compared to the wavelength of the light in the medium. In ellipsometric measurements of thin-film stacks, the reflection and the transmission coefficients of a rough surface are often calculated by replacing the rough interface by a thin film having a thickness related to the amplitude of the roughness and a permittivity derived from an appropriate EMA. In a similar manner, we approximated the roughness of a layer.

## Methods

### Materials

Silicon <100> wafers (n-type, 2-in diameter, one side polished) were chosen with resistivity 0.01 Ω. Hydrofluoric acid, nitric acid, sulfuric acid, hydrogen peroxide (without stabilizer), ethanol, and isopropyl alcohol were used throughout the experiments and used as received. A Teflon beaker was used for HF treatments. Deionized water (DI water) was used throughout the reactions.

### Sample preparation

Two-inch n-type silicon 100 wafers were cut into four quarter pieces, using a diamond wafer saw. Prior to acid treatments, silicon<100> wafers were rinsed and sonicated with ethanol, three times, followed by DI water rinsing three times for 2 min. Experiments with HF were carried out in Teflon beakers. Wafers were dipped into 1:20 vol/vol HF water for 10 s and rinsed with DI water vigorously for 5 min. After rinsing with DI water for 5 min, the surfaces were dried under nitrogen flow and stored in vacuum desiccators. The concentrations of HF were selected to achieve higher hydroxyl content SiOH on Si 100 surface after water rinsing with reasonable low oxide etching rate and low surface micro roughness. In this experiment, wafers were directly exposed to all four oxidizing acid mixtures with no prior HF dipping.

### Surface roughness

Optical Ψ and data were obtained at room temperature using a variable-angle automatic spectroscopic ellipsometer with a xenon lamp, the wave-length of which ran from 190 to 1,100 nm. The angles of incidence were chosen to be 33° and 70°, and the detector was placed at the same angle of incidence for measuring the zeroth-order diffraction.

To determine the roughness, we used a RCWA with the EMA approach, modeling the structure to two layers as a Si substrate with overlying roughness and describing the roughness using the Bruggeman EMA with dielectric functions of Si and air. Using this approach, we were able to determine independently the thicknesses of the roughness layer.

**Figure 5:**
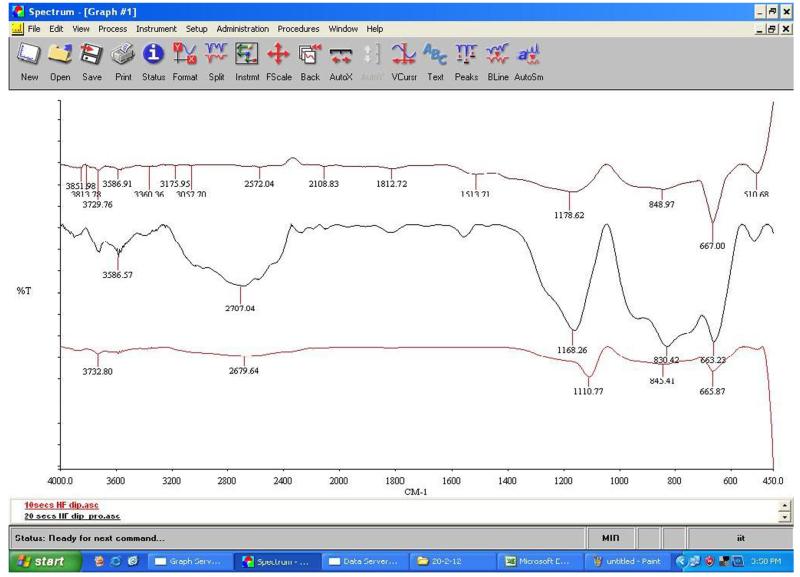
Infrared reflection spectra of 30 seconds HF treated-D.I.Water rinsed Si (100)-H surface.

The best fits to the Ψ and data are shown in Figure 5. The fittings yielded a roughness thickness of 3.3 nm for the surface. The EMA ratio of Si to air in the rough layer is 69:31, which is reasonable.

To summarize, we have shown that RCWA with EMA method can be used to assess the roughness on layers and structures.

## Results and discussion

**Figure 6:**
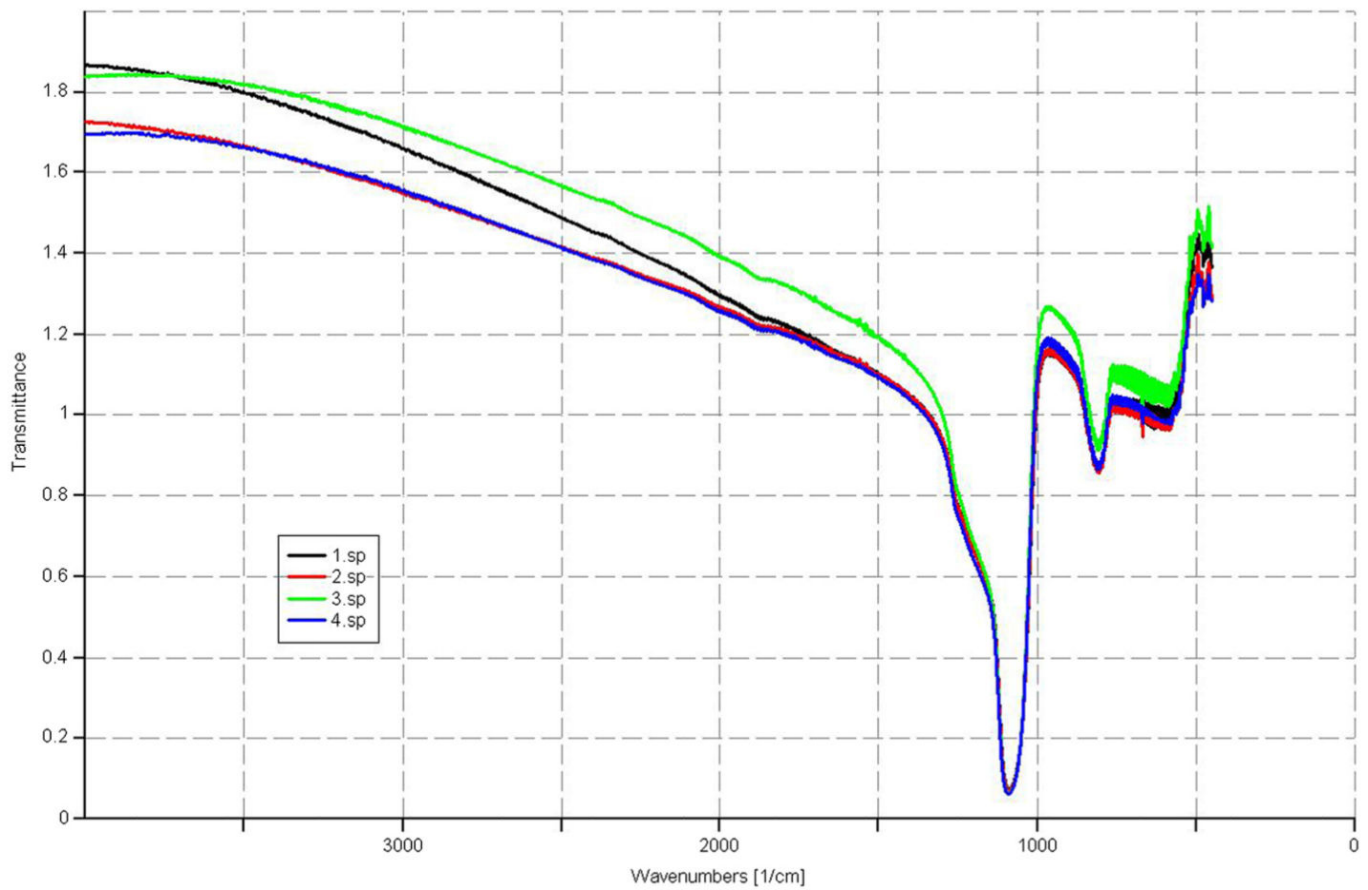
Infrared reflection spectra of Si (100)-H surface treated with diluted HF for 30 s.

**Figure 7:**
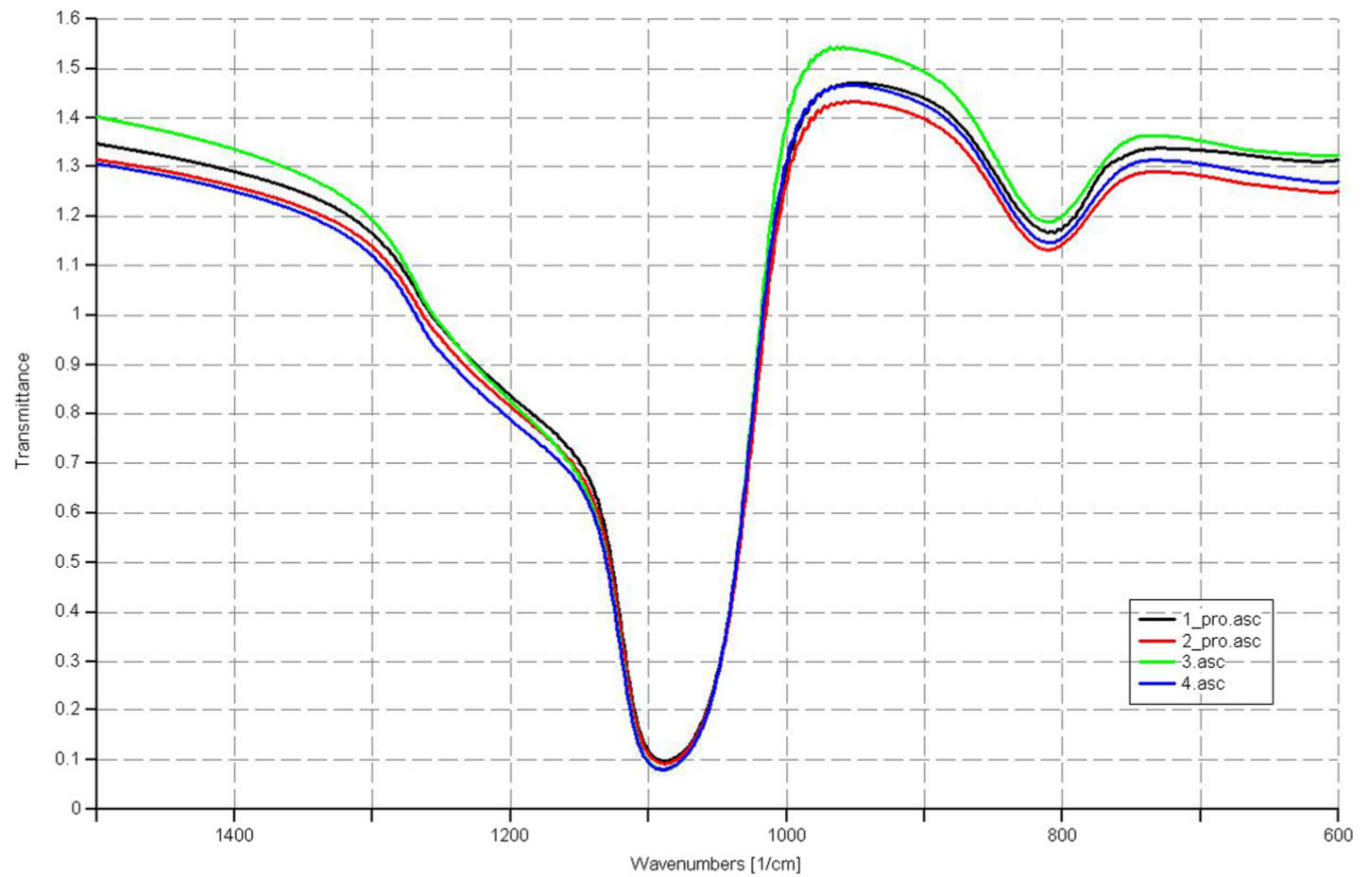
Infrared reflection spectra of Si (100) surface treated with diluted HF.

**Figure 8:**
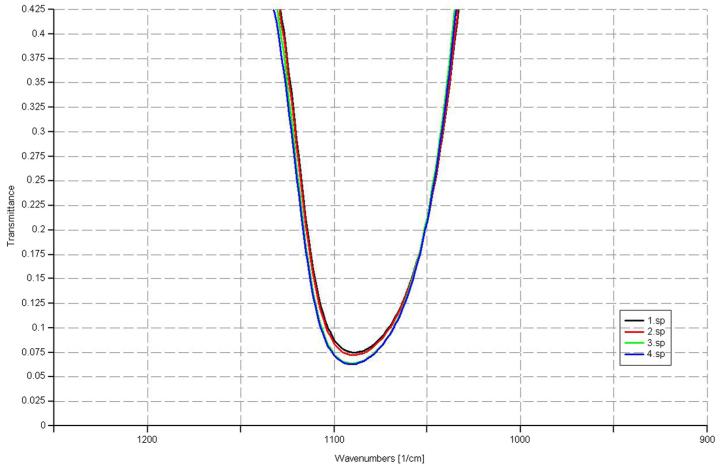
Infrared reflection spectra of Si (100) surface treated with diluted HF.

**Figure 9:**
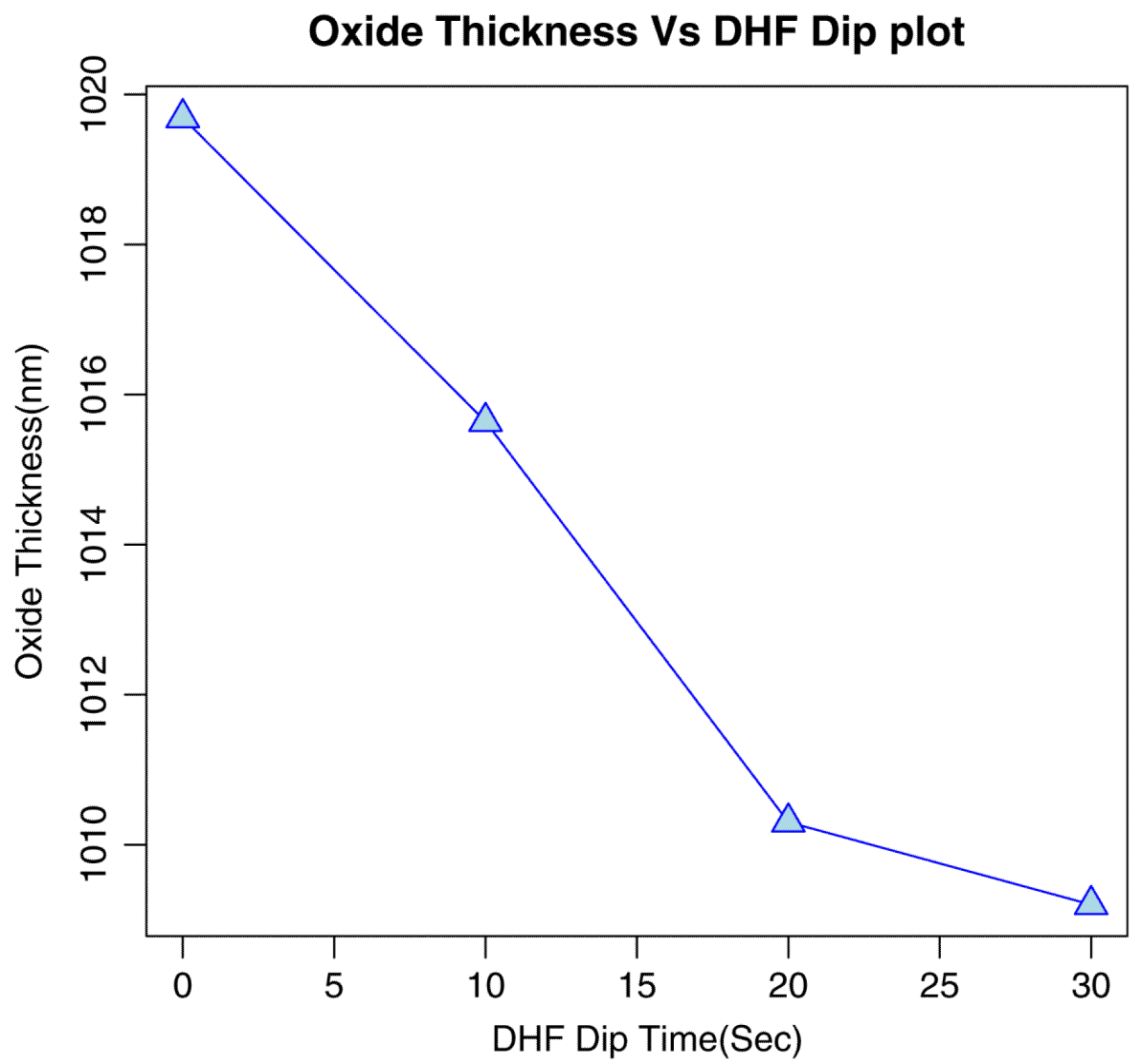
Oxide Thickness vs. DHF Dip Time plot of HF treated-D.I.Water rinsed Si (100)-H surface.

**Figure 10:**
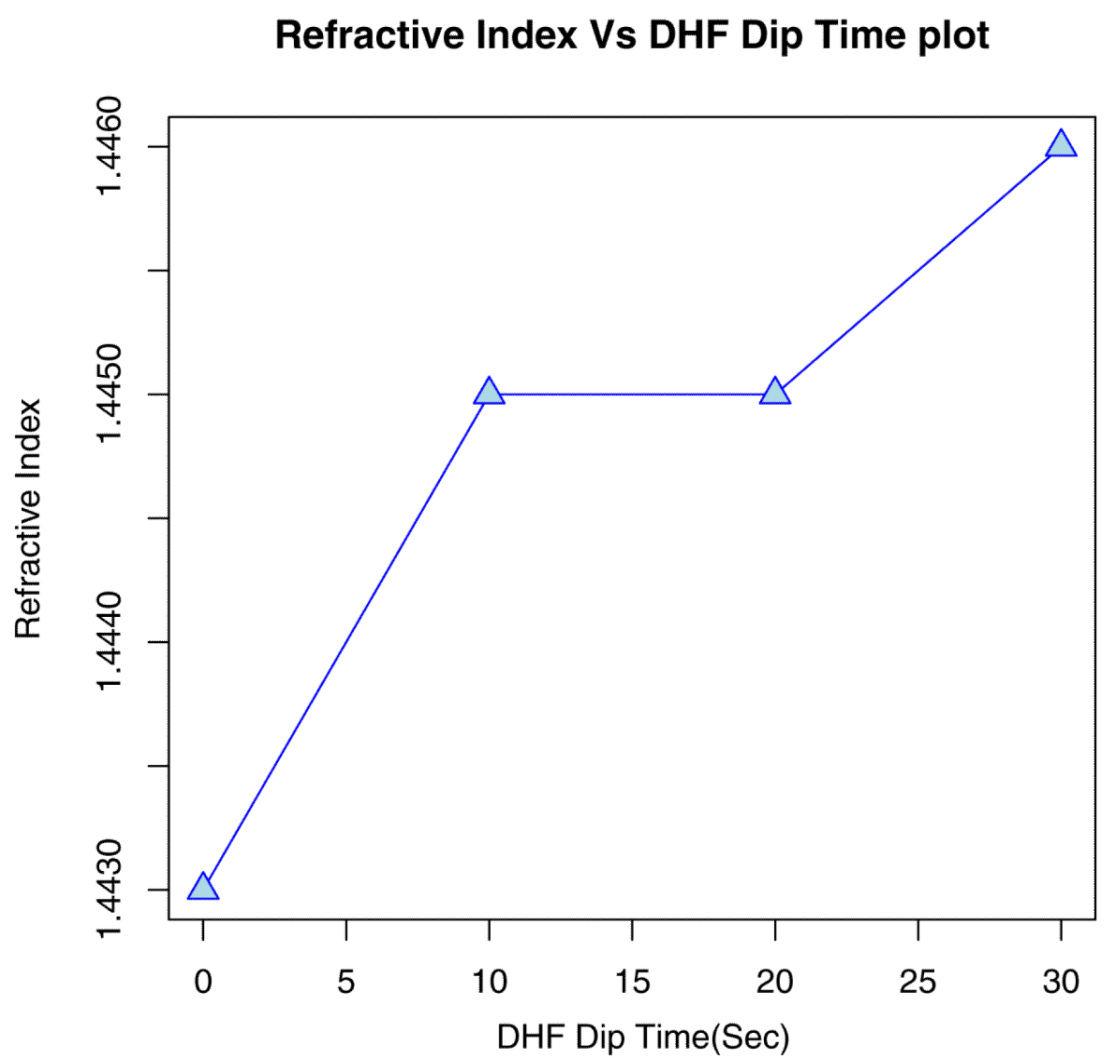
Oxide thickness vs. DHF dip time plot of dilute HF-treated Si (100)-H surface.

**Figure 11:**
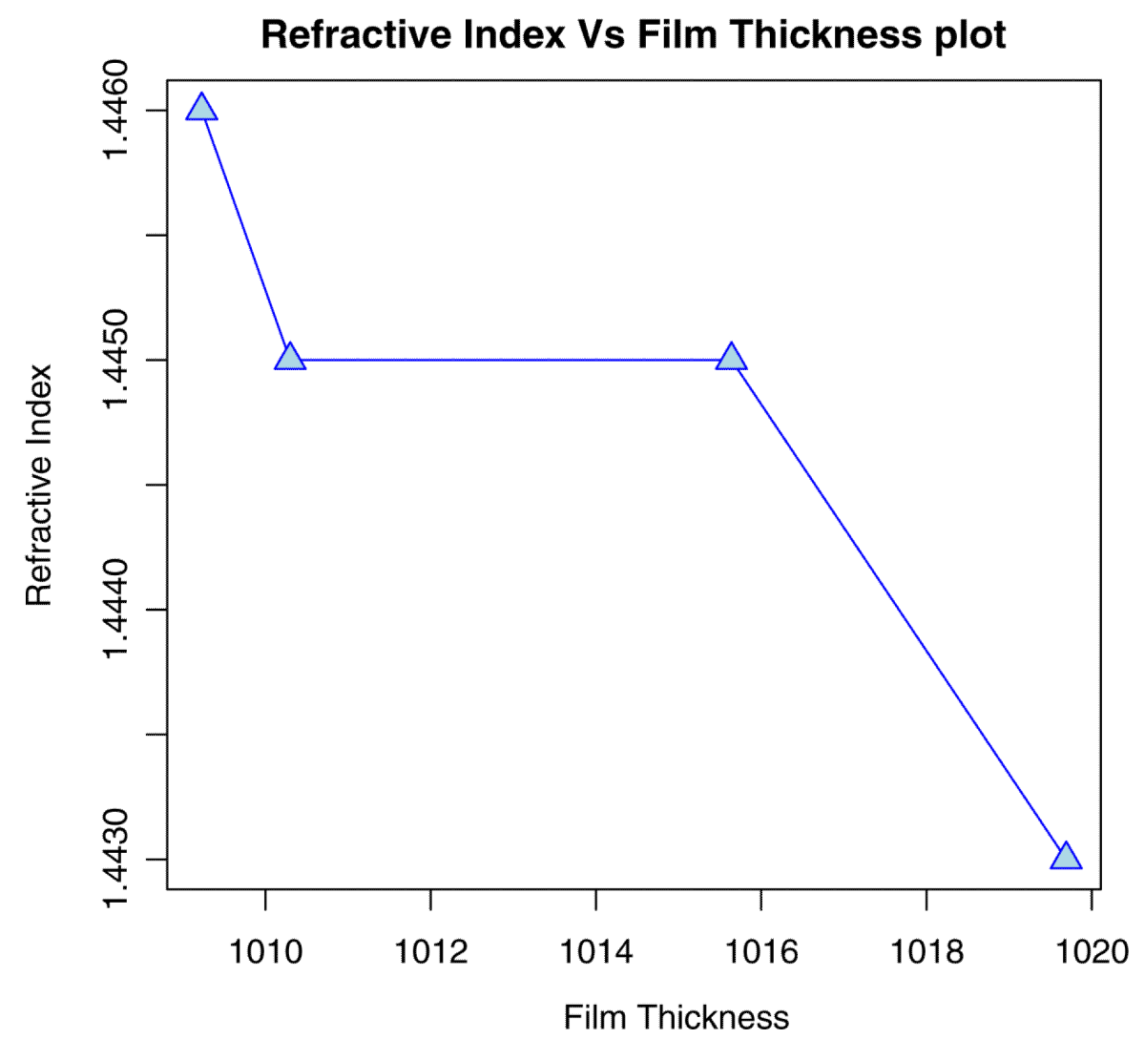
Refractive index vs. DHF dip time plot of dilute HF-treated Si (100)-H surface.

**Figure 12:**
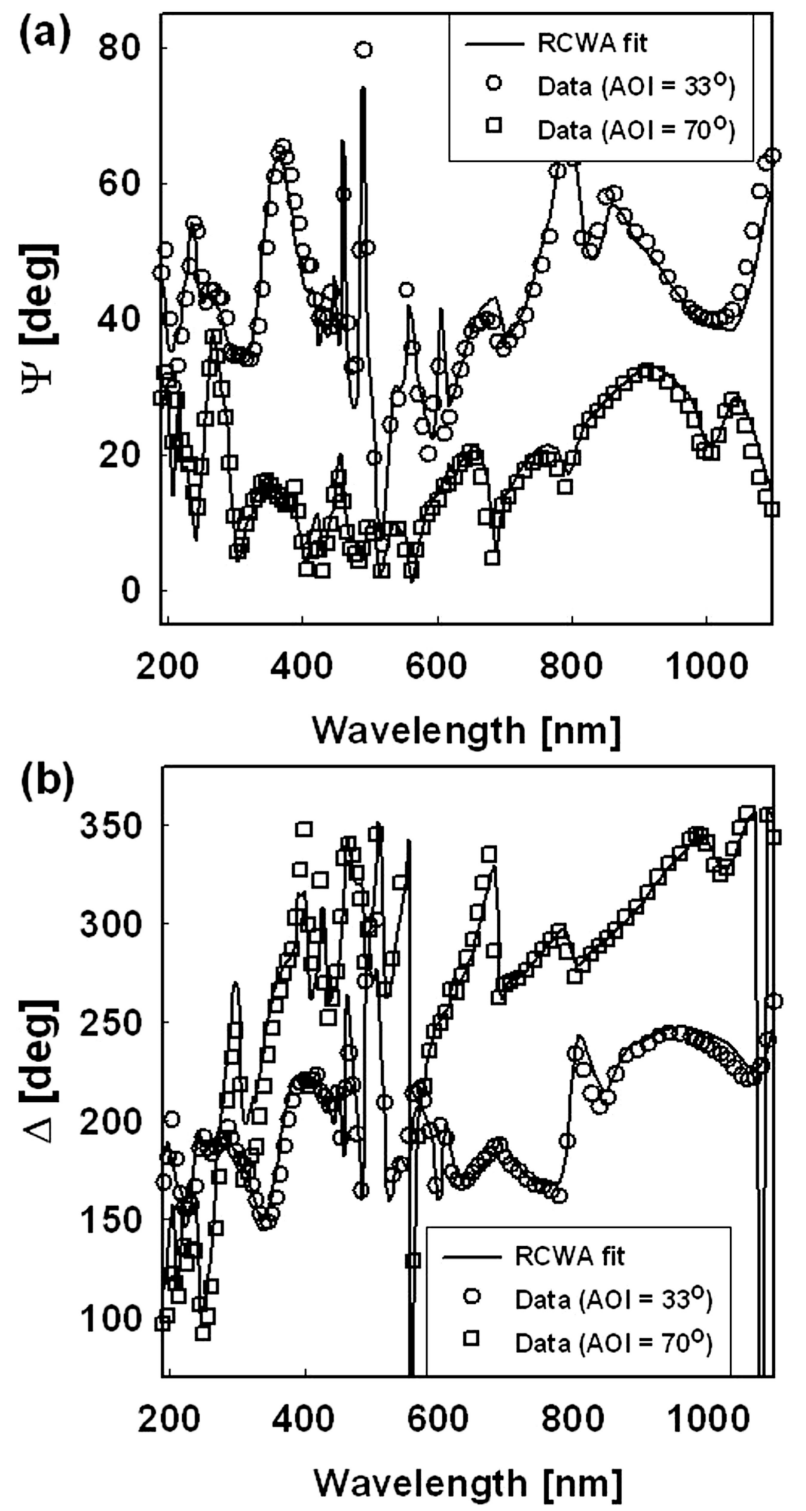
Measured (a) Ψ and (b) (circles and squares) data for the sample with best fit line determined by using the RCWA.

Using ellipsometry, we have measured the thickness of the native oxide film and the refractive index of the film as presented in Table 1. Figures 6, 7, 8, and 9 depict the typical Si-H stretching vibration spectra of the HF-treated Si-100 surface exposed to diluted HF (DHF) for 10, 20, and 30 s, respectively, collected for different exposure times. As it is shown in the top spectrum of the Figure 5, the surface immediately after HF treatment exhibits two peaks at 2,050 and 2,110 cm^−1^ which are due to the monohydride Si-SiH and dihydride Si-SiH_2_, respectively. The Si-H stretching vibration spectrum for the diluted HF-treated Si-100 surface is characterized by an intense SiH_2_ peak. This is simply due to the fact that the ideal, bare Si-100 surface has two dangling bonds per Si atom; the hydrogen termination of those dangling bonds produces the dihydride Si. When the surface was exposed to air for minutes, new peaks appeared at 2,281 and 2,250 cm^−1^, which are attributed to the intermediate oxidation states SiH_2_-O_2_ and SiH-O_3_, respectively. SiH_2_-O_2_ and SiH-O_3_ refer to atomic configurations in which, besides the hydrogen atoms, two and three oxygen atoms are bound to the surface Si atom, respectively. As seen from the FTIR spectra, the hydride modes, SiH and SiH_2_, increased in intensity with exposure time. This clearly indicates that the surface Si atoms previously having Si-H bonds were de-oxidized by exposing the surface to DHF. On the other hand, the intensities of SiH_2_-O_2_ and SiH-O_3_ initially increased and then dropped. We have previously interpreted the observed evolution in intensity of the hydrides and intermediate oxidation states as being due to the oxidation process in which four bonds around the surface Si atom, which initially were the Si-Si back bond and the Si-H bond, are converted to the Si-O bond in succession. We, therefore, confirm that oxygen is crucial to the oxidation of the topmost layer of hydrogen-terminated Si-100 surfaces as presented in Table 2. Figures 10 and 11 depicts the thickness and refractive index variations with the DHF dip time. Figure 12 depicts the film thickness vs. the refractive variation. Table 3 depicts the root mean square (RMS) noise and signal-to-noise ratio (SNR) of the spectra in the peak band and the noise band.

The FTIR spectra confirm that the DHF-dipped Si<100> has a hydrogen-absorbed layer terminating the dangling bonds. Using as low as 5% concentration of HF, we have shown the hydrophobicity of the silicon<100> surfaces.

## Conclusion

DHF dip for 20 s will convert the Si<100> surface hydrophobic because of desorption of hydrogen. The FTIR spectra confirm that the DHF-dipped Si<100> has a hydrogen absorbed layer terminating the dangling bonds. Using as low as 5% concentration of HF, we have shown hydrophobicity of the silicon<100> surfaces.

## Figures and Tables

**Table 1:** Ellipsometer measurements

Serial number	Native oxide on bare silicon	
	**Thickness**	**Refractive index**
1	1.6 nm	1.443

**Table 2:** FTIR vibration mode assignments

Serial number	Vibration modes	Sample 1 (cm^−1^)	Sample 2 (cm^−1^)	Sample 3 (cm^−1^)
1	Si-O out-of-the-plane deformation	518.95	518.95	510.8
2	Si-O bending	845.47	830.42	848.97
3	Si-OH stretching	939	939	939
4	Si-O-Si stretching	1,110.77	1,168.26	1,178.62
5	C-O bending	1,600	1,558.17	1,513.71
6	Si-C stretching	2,357	2,357	-
7	…-OH stretching	3,444	3,394	3,360
8	H_2_O Scissor mode	1,670	1,670	1,670
9	O-H, Si-OH produces from dissociative adsorption of water	3,732.80	3,727.56	3,729.76
10	Si-H (2,050 to 2,150 cm^−1^)	2,050	2,100.44	2,108.83
11	Si-O low frequency oxide (1,120 to 1,180 cm^−1^)	-	-	-
12	Si-O high frequency oxide (>1,180 cm^−1^)	1,200	1,280	1,280
13	(O1,2)Si-H (2,175 to 2,275 cm^−1^)		2,281.35	-
14	(O3)Si-H (2,225 to 3,000 cm^−1^)	2,679.64	2,707.64	2,572.04

**Table 3:** SNR and RMS noise for the wafers in the peak band and noise band

Spectra	SNR	RMS noise	Noise band start	Noise band end	Peak band start	Peak band end
1.sp	4.8970	27.6233	450	2,000	450	2,800
2.sp	4.7569	26.7883	450	2,000	450	2,800
3.sp	4.7149	30.0560	450	2,000	450	2,800
4.sp	4.7339	27.0772	450	2,000	450	2,800
